# Mechanisms of Base Substitution Mutagenesis in Cancer Genomes

**DOI:** 10.3390/genes5010108

**Published:** 2014-03-05

**Authors:** Albino Bacolla, David N. Cooper, Karen M. Vasquez

**Affiliations:** 1 Dell Pediatric Research Institute, Division of Pharmacology and Toxicology, College of Pharmacy, The University of Texas at Austin, 1400 Barbara Jordan Blvd., Austin, TX 78723, USA; E-Mail: albino.bacolla@austin.utexas.edu; 2 Institute of Medical Genetics, School of Medicine, Cardiff University, Cardiff CF14 4XN, UK; E-Mail: cooperDN@cardiff.ac.uk

**Keywords:** genetic alterations, cancer etiology, cancer genomes, functional genomics, human genome sequence, single base substitutions, DNA repair, translesion synthesis, oxidative damage, cytosine deamination

## Abstract

Cancer genome sequence data provide an invaluable resource for inferring the key mechanisms by which mutations arise in cancer cells, favoring their survival, proliferation and invasiveness. Here we examine recent advances in understanding the molecular mechanisms responsible for the predominant type of genetic alteration found in cancer cells, somatic single base substitutions (SBSs). Cytosine methylation, demethylation and deamination, charge transfer reactions in DNA, DNA replication timing, chromatin status and altered DNA proofreading activities are all now known to contribute to the mechanisms leading to base substitution mutagenesis. We review current hypotheses as to the major processes that give rise to SBSs and evaluate their relative relevance in the light of knowledge acquired from cancer genome sequencing projects and the study of base modifications, DNA repair and lesion bypass. Although gene expression data on APOBEC3B enzymes provide support for a role in cancer mutagenesis through U:G mismatch intermediates, the enzyme preference for single-stranded DNA may limit its activity genome-wide. For SBSs at both CG:CG and YC:GR sites, we outline evidence for a prominent role of damage by charge transfer reactions that follow interactions of the DNA with reactive oxygen species (ROS) and other endogenous or exogenous electron-abstracting molecules.

## 1. Introduction

The explosion of cancer genome sequencing projects over the past few years has revealed the complexity of the processes whose alterations are associated with, and are often causative of, various types of cancer [1,2]. These include mutational mechanisms that give rise to tissue-specific mutation rates, variations in mutation frequencies in distinct compartments of chromosomes, sequence context-dependent mutation spectra, and regional hypermutation, often termed kataegis (thunderstorm) [3]. Cancer genome studies have also served to catalogue the extent to which translocations, chromosomal gains and losses and focal copy-number alterations take place, often mediated by catastrophic chromosomal shattering events, as in the case of some bone and pediatric cancers [4,5,6,7,8]. From a mechanistic and therapeutic perspective, the arsenal of gene classes and pathways that are frequently altered, such as signal transduction, metabolism, DNA repair, transcription, epigenetics, RNA splicing and protein homeostasis, has also greatly expanded [1,2,9,10,11]. Attempts to address key questions concerning the causes leading to the mutational events that characterize and contribute to driving a normal cell towards tumorigenesis have also burgeoned [12,13,14]. These attempts are, however, necessarily indirect since the only material available for analysis are the catalogues of mutations that survived and accumulated, in most cases, over long periods of time. With few exceptions, such as mutation patterns observed in the lung cancers of heavy smokers and in skin cancers following UV exposure, most mutational patterns have remained enigmatic.

The goal of this review is to examine two prominent single base substitution (SBS) patterns observed in cancer genomes, both of which display sequence context-dependent signatures: C→T transitions at CG:CG (the colon separates complementary bases written in a 5'→3' direction) dinucleotide sequences and substitutions at C:G base-pairs in the context of YC:GR (Y, pyrimidine; R, purine) motifs. Whereas spontaneous deamination of 5-methylcytosine has been proposed to account for the first pattern, two mechanisms have been recently suggested for the latter pattern: over-activity by the APOBEC family of cytosine deaminases and electron transfer following oxidative damage. After considering several factors associated with SBSs, such as regional variations in mutation frequencies, mechanisms leading to base modification, and DNA repair systems, we conclude that for both SBS patterns, oxidative base damage from ROS and other electron-abstracting molecules appears to play a more significant role than previously anticipated.

## 2. Meta-Analyses of Cancer Genomes

### 2.1. Mutational Signatures in Cancer Genomes

The large number of sequenced cancer genomes now available has made it possible to address the issue of mutational spectra and relative mutation frequencies, both exome-wide and genome-wide across different cancer types. Some of the largest meta-analyses have included 4,938,362 somatic substitutions and small insertions/deletions (indels) from 7,042 primary cancers of 30 different classes [13], ~1,000,000 somatic exome mutations from 4,800 tumors representing 19 different cancers [15], 617,354 somatic mutations in 3,281 tumors from 12 cancer types [16], 533,482 somatic SBSs from 1,149 cancer samples and 2 cell lines representing 14 different tissues [17], and 373,909 non-silent coding mutations in 3,083 tumor-normal pairs across 27 tumor types [18]. The prevalence of SBSs was highly variable, both between and within cancer classes, ranging from ~0.001 per megabase (Mb) to >400 per Mb, with childhood cancers generally carrying the fewest mutations, acute myeloid leukemia exhibiting a very low median mutation frequency (~0.28/Mb), and cancers associated with chronic mutagen exposure, such as lung (tobacco smoking) and malignant melanoma (UV light) displaying the highest mutation frequencies (8.15/Mb for lung squamous cell carcinoma) [13,15,16].

With regard to mutational spectra, the most consistent and frequent mutational signature across cancer types has been noted at CG:CG (we identify both nucleosides and nucleotides by their base) dinucleotides, with 25/30 cancer types in [13] and 13/14 in [17], and gastrointestinal tumors displaying CG:CG→TG:CA (target base underlined) among the highest fractions [13,16,17] (Table 1A). The preponderance of C→T transitions at CG:CG sequences has been attributed to high rates of spontaneous deamination of 5-methylcytosine (5mC) as compared to unmethylated cytosine [13,16,17,19]; such deamination events yield T:G mismatches and, subsequently, G→A transitions at the next round of DNA replication. A second prominent mutational signature found across several cancer types, including breast, ovary, bladder, head and neck, cervix, liver and lung [13,15,16,17,20,21], has been noted at C:G base-pairs in the context of TC:GA dinucleotides (C→T, C→G and C→A) (Table 1A); this has been attributed either to over-activity of members of the apolipoprotein B mRNA-editing catalytic polypeptide (APOBEC) cytosine deaminases [13,15,18,20], or to electron transfer reactions following oxidative damage [17].

Cancer type-specific mutational signatures have also been identified, particularly in cancers of the lung and skin. For example, in a cohort of 17 non-small cell lung cancer patients, the total number of somatic mutations was ~10-fold higher in smokers (median 15,659, range 7,424–26,202) than in never-smokers (median 888, range 842–1,268) [22], consistent with other reports that smoking-associated lung cancer is distinguished by a significantly high number of mutations per Mb [23,24,25,26,27,28]. Tumors from smokers were also characterized by high fractions (up to 46%) of C:G→A:T transversions [22,27,28,29,30] (Table 1B), a signature of exposure to alkylating nitrosamines and polycyclic aromatic hydrocarbons (PAHs) present in tobacco smoke, which yield miscoding G adducts [31,32,33,34]. This conclusion is further supported by a recent whole-genome sequencing analysis of an arsenic exposure-related lung squamous cell carcinoma, which was instead characterized by a high fraction (16.3%) of T:A→G:C transversions but a low fraction (~6.1%) of C:G→A:T transversions [35] (Table 1B). In melanomas, up to 87% of all mutations were represented by C:G→T:A transitions, mostly at pyrimidine dimers [36,37,38,39,40,41,42], consistent with DNA translesion synthesis across UV-induced covalently linked pyrimidine dimers [43]. Mutations in skin cancer at pyrimidine dimers, particularly CC→TT transitions on the non-transcribed strand of expressed genes, have also been attributed to APOBEC3A, a member of the APOBEC family of cytosine deaminases which are active mostly on single-stranded DNA, and expressed in skin keratinocytes [44] (Table 1B). Other prominent patterns included T→C transitions in hepatocellular carcinomas, which have been attributed to bulky DNA adducts on adenine, A→T transversions in the TA:TA context, particularly in leukemia samples [13,18], a 2-fold increase in mutations at NGRA relative to NGRB (B = C or G or T) in melanomas [17] (Table 1B) and several others, for which the underlying mechanisms remain unknown.

**Table 1:** Mutational signatures in cancer genomes.

A. Main mutational signatures revealed from meta-analyses of cancer genomes				
Total number of mutations	Total number of cancer types	Major SBS signature (% cancer types)	Sequence context	References	
4,938,362	30	C:G→T:A (80%)	NCG:CGN	[13]	
		C:G→T:A or G:C (50%)	TCN:NGA		
1,000,000	19	C:G→any subst. (32%)	TCN:NGA	[15]	
617,354	12	C:G→T:A (33%)	CG:CG	[16]	
		C:G→G:C (25%)	TC:GA		
533,482	14	C:G→any subst. (93%)	NNCG:CGNN	[17]	
		C:G→any subst. (36%)	NYCH:DGRN		
373,909	27	C:G→T:A (30%)	CG:CG	[18]	
		C:G→any subst. (11%)	TC:GA		
**B. Main cancer type-specific mutational signatures**				
**Cancer type**	**Putative cause**	**SBS signature**	**Sequence context**	**References**	
Lung cancer	tobacco smoke	C:G→A:T	none	[22,27,28,29,30]	
	arsenic exposure	T:A→G:C	none	[35]	
Melanoma	UV, APOBEC3A	C:G→T:A	pyrimidine dimers	[36,37,38,39,40,41,42,44]	
	unknown	G:C→any subst.	NGRA:TYCN	[17]	
Liver carcinoma	carcinogens	T:A→C:G	none	[13,18]	
Leukemia	unknown	A:T→T:A	TA:TA	[13,18]	
Endometrial cancer	POLE^P286R^	G:C→T:A	AGA:TCT		

*N*, any nucleotide; *Y*, C or T; *R*, A or G; *D*, A or G or T; *H*, A or C or T.

### 2.2. Mismatch Repair and DNA Replicative Polymerase Proofreading Genes

The high fidelity of human DNA replication achieves nucleotide incorporation error rates of ~10^−9^–10^−1^^0^ in part through the proofreading (3'→5' exonuclease) activities of replicative polymerases, Pol ε and Pol δ, and postreplicative mismatch repair (MMR), which decrease the rates of misincorporation on the newly synthesized daughter strands by 100–1,000-fold each [45,46,47,48]. The proofreading domains reside in the large 261 and 125 kDa POLE and POLD1 catalytic subunits of the Pol ε and Pol δ holoenzymes, respectively, which perform their specific function predominantly during leading-strand (Pol ε) and lagging-strand (Pol δ) DNA synthesis in S phase [49]. The MMR pathway comprises 6 genes (*MSH2*, *MSH3*, *MSH6*, *MLH1*, *PMS1* and *PMS2*) whose products yield 4 types of heterodimeric complexes [MutSα (MSH2/6), MutSβ (MSH2/3), MutLα (MLH1/PMS2) and MutLβ (MLH1/PMS1)], active on mismatches, bulges, small loops, and a number of DNA lesions [50,51].

More than 1,000 constitutional gene variants in *MLH1*, *MSH2*, *MSH6* and *PMS2* have been classified as pathogenic or likely pathogenic in patients affected by Lynch syndrome [52], an autosomal dominant condition also known as hereditary non-polyposis colorectal cancer (HNPCC) and characterized by increased susceptibility to colorectal (25%–70%), endometrial (30%–70%), and other types of cancer [53]. Such MMR defects have also been known to lead to an accumulation of mutations, mostly in the form of microsatellite length changes (microsatellite instability, MSI) [53]. The patterns of SBSs in cancers showing MSI have recently been addressed from the exome and whole-genome sequencing data of two large cohorts of colorectal [54] and endometrial [55] cancer patients (224 and 373 tumors, respectively), and the reconstructed whole-genomes from two gastric cancer patients [56]. In general, there was no relationship between MSI status (high/low) and SBS mutation rates. By contrast, most samples with elevated SBS mutation rates also displayed somatic mutations in the proofreading *POLE* domain [57]. In addition, *POLE*-mutated samples could be classified into two distinct groups based on *MLH1* status: group 1, with low SBS mutations rates, *MLH1* inactivation and MSI-high; and group 2, with high SBS mutation rates, functional *MLH1* and MSI-low. Thus, paradoxically, concomitant *POLE* and *MLH1* mutations do not appear to act synergistically on SBS mutation rates in most patients [57].

Mutations in the proofreading domains of *POLE* and *POLD1* were also reported in two human colorectal cancer cell lines (DLD-1 and LoVo) and 1/76 colorectal cancer patients, all three samples exhibiting MMR deficiency [58]. More recently, two recurrent germline mutations in the proofreading domains of *POLE* (L424V) and *POLD1* (S478N) have been found in a cohort of 3,805 colorectal cancer patients selected for family history of colorectal tumors and multiple adenomas [59]. In the 62 tumors analyzed, no MSI was found; rather, loss of heterozygosity, chromosomal instability and driver mutations in known cancer genes, including *KRAS*, *BRAF*, *APC*, *PIK3CA*, *FBXW7*, but not *CTNNB1*, were revealed, suggesting that mutant POLE^L424V^ and POLD1^S478N^ may promote tumor formation by increasing the rates of SBS, without any apparent bias for a predominant type of base substitution [59]. Twelve missense somatic mutations predicted to affect the proofreading domain of *POLE*, and all associated with microsatellite stability, have also been identified in a study of 173 endometrial cancers, P286R being the most commonly represented (6 times) POLE mutation [60]. Using a panel of 75 cancer genes, *POLE*-mutated tumors exhibited an ~6-fold increase in mutations relative to non-*POLE*-mutated tumors, with a prevalence of G:C→T:A transversions, particularly at G:C base-pairs flanked 5' and 3' by an A:T base-pair [60]. Further validation of the putative role for *POLE*, and to a lesser extent *POLD1*, mutations in endometrial cancer has been obtained from an analysis of unpublished TCGA genomic data, where 21 (8.5%) and 1 (0.4%) tumors out of 248 samples were found to harbor *POLE* and *POLD1* mutations, respectively, including 8 cases of P286R and 5 cases of V411L changes in POLE [60]. *POLE/POLD1*-mutated cancers displayed high SBS rates, ranging from 227 to 14,695 exonic events, compared to a range of 22 to 2,014 in cancers lacking *POLE/POLD1* mutations. Likewise, cancers carrying the POLE^P286R^ allele exhibited an overrepresentation of G:C→T:A substitutions at AGA:TCT motifs (Table 1B), supporting a DNA sequence-specific proofreading defect for this particular POLE mutation [60]. In summary, germline and somatic mutations in the *POLE* and *POLD1* genes appear to predispose to, or promote, colorectal and endometrial cancers in part by increasing the rates of SBSs [61]. Less direct investigations from cell culture nuclear extracts suggest that defects in DNA replication fidelity might also be associated with ovarian cancers [62].

### 2.3. The APOBEC Family of Cytosine Deaminases

The family of APOBEC cytosine deaminases comprises eleven members with distinct functions: activation–induced deaminase (AID), a B cell-specific enzyme required for both somatic hypermutation (SHM) and class-switch recombination (CSR); APOBEC1, which is expressed primarily in the gastrointestinal compartment and is active in the transcript sequence editing of the apolipoprotein B mRNA; APOBEC2, which is expressed in heart and skeletal muscles and which appears to be essential for muscle development; APOBEC3s (A, B, C, D, F, G and H), which are active against exogenous viruses and endogenous retroelements and hence important for innate immunity; and APOBEC4, which is mostly expressed in the testes but whose function remains unknown [63]. APOBECs have in common a zinc-dependent cytidine deaminase domain (ZDD), which catalyzes the conversion of cytosine and deoxycytidine to uracil and deoxyuracil [64] in single-stranded RNA and DNA, often in a sequence-dependent context.

Editing activities of APOBEC3s play a critical role in restricting viral infectivity, and have also been postulated to have counteracted the actions against genome stability, mostly in terms of integration, exerted by both non-LTR (long terminal repeats) and LTR retrotransposons during evolutionary time [63,65]. For example, in Δvif (virion infectivity factor) HIV-1 particles, APOBEC3G proteins interact with the nucleocapsid domain of viral Gag to form nucleoprotein complexes with several Pol-II and Pol-III transcribed RNAs, which are then encapsulated into virions. During HIV-1 reverse transcription, up to 10% of cytosines can be deaminated to uracil on the minus strand of the viral complementary DNA (cDNA), thereby promoting loss of genetic information and the production of large populations of defective virions [63]. Vif antagonizes the activity of APOBEC3G (and other APOBEC3s) by binding and targeting the enzyme for polyubiquitination and subsequent degradation [63,66,67]. Other viruses targeted by APOBEC3 enzymes include human T-cell lymphotropic virus (HTLV), hepatitis B virus (HBV), hepatitis C virus (HCV), human papillomavirus (HPV) and human herpesviruses (HHV). In addition to deaminase activity, APOBEC enzymes restrict exogeneous viruses and endogenous retroelements through editing-independent activities. These include inhibition of viral replication, for example by interfering with tRNA priming and the initiation of DNA replication during HIV-1 reverse transcription, binding with positive regulators of viral gene expression, as in the case of heterogeneous nuclear ribonucleoprotein K (hnRNP) for HBV [63], and other less well-characterized mechanisms aimed at restricting non-LTR retrotransposon transcription, DNA synthesis and integration [65].

At least three recent reports have suggested the involvement of aberrant APOBEC3B deaminase activity as a frequent cause of SBSs in cancer. For example, APOBEC3B mRNA was found to be upregulated relative to controls in 28/38 established breast cancer cell lines and to be expressed in the nucleus [68]. In selected nuclear extracts, C→U editing activity was detected on synthetic DNA substrates specifically at TC:GA dinucleotides when treated with a control shRNA, whereas no activity was evident upon treatment with short hairpin RNA (shRNA) targeting APOBEC3B mRNA. In these cell lines, treatment with anti-APOBEC3B shRNA also led to a decrease in genomic uracil loads from ~100,000 to ~60,000 per haploid genome by HPLC-ESI-MS/MS and, as expected, test amplicons displayed a decrease in C→T transition mutation frequency. The involvement of APOBEC3B activity in cancer was further supported by data on Ref-seq APOBEC3B expression, which was shown to be high in several tumor types, including breast, uterus, bladder, head and neck and lung (both adenocarcinoma and squamous cell carcinoma) [15,20]. The top five cancer types with the majority of mutations at C:G base-pairs were also among the top six datasets in terms of APOBEC3B mRNA expression, and a positive correlation between the proportion of mutations at C:G base-pairs and median APOBEC3B levels was observed. Bladder, cervical, lung squamous cell carcinoma, lung adenocarcinoma, head and neck, and breast cancers shared a strong bias for TCN (N = A or C or G or T) mutation signatures, as observed for the recombinant APOBEC3B protein. Interestingly, a significant enrichment of strand-coordinated and clustered (2 or more per 10 kb) C→T and C→G mutations at TCW (W = A or T) motifs were discovered, a number of which were in close proximity to chromosomal rearrangement breakpoints, particularly in bladder, cervical, head and neck, breast and lung tumors [15,20], a phenomenon termed kataegis [3]. In summary, these analyses are consistent with the possibility that aberrant APOBEC deaminase activity, particularly at TC:GA sites, may represent a general endogenous mutagen that contributes to several different types of human cancer.

### 2.4. Electron Transfer in DNA Oxidation

Mutation spectra analyses of SBSs arising spontaneously, both in cell culture and in whole animals, have indicated the frequent occurrence of sequence context-dependent mutations. For example, an analysis of 837 spontaneous SBSs in the *supF* tRNA gene in 18 cell lines and 2 transgenic mouse models indicated that the most mutable regions involved guanine and cytosine tracts [69]. In human osteosarcoma cells, shRNA knock-down of the *WRN* helicase gene, mutations in which are associated with the progeroid Werner syndrome, led to a doubling in genomic 8-oxo-7,8-dihydro-2'-deoxyguanosine (8-oxoG) content and an increase in SBSs in the *supF* reporter gene, consisting largely of G→C (49%), G→A (28%) and G→T (23%) substitutions at GA:TC dinucleotides within the *supF* reporter gene [70]. Because 8-oxoG is a well-recognized marker of oxidative damage and guanine oxidation depends upon flanking sequence (*i.e.*, GR > GY; R = A or G; Y = T or C) as a result of stacking-induced electron transfer [71,72], these data suggested a role for oxidative damage in sequence context-dependent mutagenesis.

A recent study addressed the question as to whether electron transfer might also cause sequence context-dependent SBSs in cancer [17]. The analysis compared the fractions of mutations occurring at G:C base-pairs in the context of all 64 possible combinations of NGNN:NNCN motifs (NGNN for simplicity), and included 21 cancer datasets representing 14 tissues comprising 1,149 patient samples and 2 cell lines for a total of 533,482 SBSs. With the exception of two melanoma datasets, CGNN sequences were more frequently mutated than DGNN (D = A or G or T), consistent with the CG:CG dinucleotide, the most prominent substrate for cytosine methylation, being a common mutation hotspot (Table 1A). In 7 cancer datasets, including lung, head and neck and melanoma, for which association with exposure to either cigarette smoke or sunlight was documented, G followed by a 3' purine was associated with increased mutations as compared to a 3' pyrimidine, *i.e.*, DGRN > DGYN (Table 1A). Notably, significant correlations were observed between the fractions of mutated DGNN motifs and the sequence-dependent free energies of base stacking along the DGNN motifs for 5 of the 7 cancer datasets. Significant correlations were also observed between the fractions of mutated DGNN motifs and the energies required to abstract an electron from the target guanines, as assessed from the values of vertical ionization energies computed for all G-centered trimer motifs. These results are consistent with the conclusion that DNA oxidation may be a source of sequence context-dependent SBSs in cancer as a result of electron transfer, as postulated from model sequences *in vitro* [73,74]*.*

### 2.5. DNA Replication Timing

During eukaryotic DNA replication, more than 20,000 pre-replicative complexes comprising the origin recognition complex (ORC), Cdc6 and Cdt1 load inactive minichromosome maintenance (MCM) helicase complexes to generate “licensed” replication origins. Initiation of DNA synthesis is controlled by Dbf4-dependent kinase (DDK), which recruits Cdc45 and Sld3, and cyclin-dependent kinase (CDK), which by phosphorylating Sld3 and Sld2 recruit GINS and additional factors, including DNA polymerases. Origin firing is controlled both temporally and spatially. Chromatin correlating positively with gene expression, G + C-richness and active chromatin marks is replicated in early S phase in the nuclear interior, whereas chromatin associated with gene-poor regions, A + T-richness and repressive chromatin marks is preferentially replicated during late S phase at the nuclear periphery [75,76].

In cancer, late replicating chromatin has been shown to harbor higher relative fractions of SBSs than early replicating chromatin. For example, the fractions of SBSs from several completely sequenced cancer genomes (melanoma, prostate cancer, small cell lung cancer, chronic lymphocytic leukemia and colorectal cancer) were found to be significantly more extensive in the constant late than in the constant early replicating zones of neutrally evolving regions [77]. Such genomic regions were those remaining after sequences from centromeres, telomeres, the Y chromosome, genes, promoters, repetitive elements and ultra-conserved regions had been excluded. Analyses of the mutation spectra indicated that, with the exception of A→T (T→A) transversions, which occurred more often in the constant late replicating regions in all five cancer types, the relative proportions of substitutions were very similar between constant early and constant late replicating regions, even though mutation frequencies were higher in the latter. Similarly, a large study of SBSs in exomes from 3,083 tumor-normal pairs representing 27 different cancer types also found that the average mutation fraction was higher (~2.9-fold) in the latest- as compared to the earliest-replicating percentiles [18].

The increased mutation frequency in late as compared to early replicating regions does not appear to be a unique property of cancer cells. For example, a comparison of 1-Mb non-overlapping regions containing SBSs between human and chimpanzee and the pooled cancer data [77] indicated that most regions harboring human-chimpanzee SBSs also harbored SBSs in cancer. Similarly, in human populations, late-replicating regions of the human genome have been shown to be characterized by a greater density of single nucleotide polymorphisms (SNPs) than early replicating regions [78]. Finally, deep-sequencing of human lymphoblastoid cell lines from father-mother-offspring trios revealed that transition mutations were >2-fold more abundant in late-replicating than in early-replicating regions of the genome, whereas transversion mutations were increased >6-fold [79]. In summary, these analyses suggest that the increased mutation rate in late- *versus* early-DNA replicating regions, which has been noted in both population and cancer genome studies, are potentially caused by mechanisms that share some commonalities between the germline and the soma.

### 2.6. Chromatin Organization

Chromatin structure has been found to strongly correlate with regional SBS rates along chromosomes in cancer genomes. Chromatin organization is regulated by many factors, including epigenetics, *i.e.*, reversible changes both at the level of DNA and involving histone tail amino acids. Chromatin condensation, or heterochromatin, is associated with reduced gene expression and involves the accumulation of specific histone marks, including methylation of lysines 9 and 27 on histone 3 (H3K9me2 and H3K27me3) by histone methyltransferases (HMT) such as G9a, GLP and SETBD1, and DNA methylation at C5 of cytosine, mainly at CG:CG dinucleotides, by DNA (cytosine-5)-methyltransferases (DNMT1, DNMT3a and DNMT3b). Among other critical interactions, H3K9me2 serves as a high-affinity binding site for the recruitment of heterochromatin protein 1 (HP1), a platform protein that collapses chromatin into higher-order fibers as a result of dimerization between nucleosomes. By contrast, promoter DNA demethylation and acetylation of H3K9 maintains open chromatin structure, or euchromatin, and supports active gene transcription [80]. These represent only a few epigenetic marks that are known to influence chromatin structure.

A total of 84,879 unique SBS positions from leukemia, melanoma, small cell lung cancer and prostate cancer genomes were used to identify potential sources of mutation rate variation across the genome, by correlating site variation with a set of 46 diverse genetic and epigenetic features that had been mapped genome-wide in human cells [81]. On a megabase scale, cancer SBS density correlated with many features of somatic cell chromatin organization, with the highest positive correlations being represented by the repressive histone modification H3K9me3, followed by H3K9me2 and H4K20me3. In fact, >55% of the variance in cancer SBS regional variation could be accounted for by combining features, with H3K9me3 alone associating with more than 40% of the observed variance in SBS density. Interestingly, the associations remained strong when only non-genic or only genic regions of the genome were considered, suggesting that transcription or transcription-coupled nucleotide excision repair may have played a comparatively minor role as compared to epigenetic modifications. We should note that, in a separate study, levels of gene transcription were found to correlate inversely with mutation rates [18], although the process of transcription is known to be a source of genetic instability [82,83]. The use of a metric that employed data on physical contacts between regions through three-dimensional folding of chromosomes, thereby distinguishing between densely packed chromatin with strong short-range interactions and accessible euchromatin with more diverse interactions, also revealed an anti-correlation pattern with somatic SBS density [81]. These findings led the authors to propose that chromatin organization is a major determinant of variation in regional mutation rates in cancer. Specifically, SBS rates in cancer cells appear to be highest in inaccessible, heterochromatin-like regions and lowest in accessible euchromatin-like domains. Reversible histone acetylation and deacetylation events in the cell are also critical for mutation avoidance. For example, failure to deacetylate H3K59ac marks following the S-phase in *hst3*Δ *hst4*Δ double-mutant yeast cells increased the rates of SBS ~10-fold and the rates of gross chromosomal rearrangements 15,600-fold, whereas lack of H3K59 acetylation in *rtt109*Δ cells led to a ~10-fold increase in complex mutations [84]. These effects were synergistic with mutations in MMR and the proofreading activities of replicative Pols δ and ε, suggesting a model in which cyclic acetylation and deacetylation of chromatin is critical for replication fidelity.

## 3. Mechanisms of Base Modification

### 3.1. Cytosine Methylation and Demethylation

**Figure 1:**
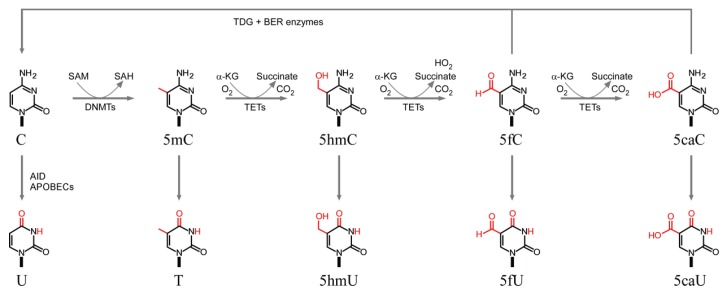
(**Top**) Cytosine methylation and demethylation pathways; (**Bottom**) Products of cytosine and C5-substituted cytosine deamination.

The ability of site-specific DNA (cytosine-5)-methyltransferases to transfer the methyl group from *S*-adenosylmethionine to the C5 position of cytosine in the context (mainly) of CG:CG dinucleotides in mammals and the role of DNA methylation in transcriptional regulation, genomic imprinting and silencing of repetitive DNA have been well reviewed [85,86]. During the past few years, it has become evident that DNA methylation can be reversed by a group of enzymes belonging to the ten-eleven translocation (TET1, 2, and 3) family of iron and α-ketoglutarate (α-KG)-dependent dioxygenases, which utilize molecular oxygen to transfer a hydroxyl group to 5mC to form 5-hydroxymethylcytosine (5hmC) (Figure 1, top). Whereas approximately 4% of all cytosines (70%–80% of CG:CGs) are estimated to be methylated, only ~0.1%–0.7% total appear to be marked by hydroxymethylation [87]. TET enzymes can further oxidize 5hmC sequentially to yield 5-formylcytosine (5fC) and 5-carboxycytosine (5caC), and evidence is increasing for a role of thymine DNA glycosylase (TDG), a member of the base excision repair pathway, in cleaving 5fC and 5caC thereby yielding abasic sites which are then replaced with unmodified cytosines [88,89] (Figure 1, top).

A critical step in the TET-dependent oxidation reactions is the role played by α-KG, which binds to a His-His-Asp-coordinated Fe(II) cluster in the enzymatic active site to effect the transfer of an oxygen to the substrate 5mC and release 5hmC. α-KG is synthesized from isocitrate in a fully reversible reaction by different isoforms of NADP^+^-dependent isocitrate dehydrogenases (IDH), with IDH2 and IDH3 acting in the mitochondria as part of the tricarboxylic acid (TCA) cycle (Krebs cycle), and IDH1 providing a source of NADPH in the cytoplasm for lipid biosynthesis and protection from oxidative stress [90].

**Figure 2:**
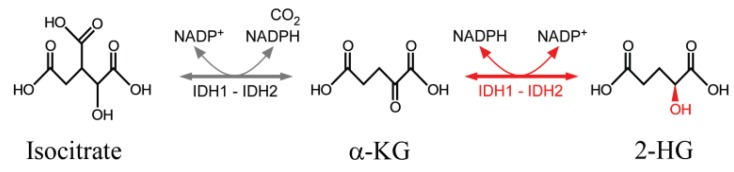
Conversion of isocitrate to α-ketoglutarate (α-KG) by isocitrate dehydrogenases (IDH) enzymes and conversion of α-KG to d-2-hydroxyglutarate (2-HG) by gain-of-function mutations in *IDH1* or *IDH2*.

Recurrent gain-of-function mutations in *IDH1* and/or *IDH2* typify ~70% of sporadic high-grade gliomas and secondary glioblastomas, ~10% of acute myeloid leukemias [16] and colangiocarcinomas [91], and have been reported in patients with acute lymphoblastic leukemia, chondrosarcomas, angioimmunoblastic T-cell lymphoma, cholangiocarcinoma and pancreatic cancers [90,92], where they have been found to induce DNA hypermethylation at CG:CG islands and shores in a tissue-specific manner [91]. The mutations, which occur at the active site of IDHs, alter the reaction order of the enzymes such that high concentrations of d-2-hydroxyglutarate (2-HG), rather than α-KG, are released [92] (Figure 2). Thus, 2-HG binding to both TET enzymes as well as other cellular dioxygenases, including histone demethylases and propyl hydroxylases, effectively inhibits their activities.

In melanoma, loss of 5mC through oxidation to 5hmC has been observed both as a result of *IDH1* or *IDH2* neomorphic mutations as well as the downregulation of *TET* and *IDH2* genes [93]. A comparison between benign nevi and melanoma further supported the selective loss of 5hmC in melanoma; in addition, the extent of 5hmC loss in melanoma correlated directly with Breslow depth, a predictor of prognosis, pathological stage and, most significantly, Kaplan-Meier survival curves [93]. Large losses of 5hmC peaks and higher levels of 5mC in melanoma *versus* nevi were detected within gene coding and flanking regions, including genes associated with adherens junctions, Wnt signaling, additional pathways in cancer, and melanogenesis pathways, implying a role for 5hmC in pathways that are fundamental to cellular differentiation and dedifferentiation. In mouse embryonic stem cells, 5fC and 5hmC were found to be enriched in intragenic regions, especially within exons and enhancers, where they colocalized with histone acetyltransferase p300 sites, DNaseI hypersensitive sites and CTCF-bound regions, specifically at poised enhancers, which are marked by H3K4me1[+] and H3K27ac[–], in comparison to active enhancers (H3K4me1[+] H3K27ac[+]), concomitantly with a decrease in 5mC [88]. Accordingly, in the absence of TDG, accumulation of 5fC correlated with increased binding of the transcriptional activator p300 at poised enhancers. These data support a role for 5mC and 5hmC oxidation in the regulation of the epigenetic state of functional enhancer elements in mammalian genomes. CG:CG hypermethylation at specific genes was also found to represent a marker for relapse-free survival time after surgery in a cohort of 444 patients with non-small cell lung cancer. Specifically, patients with zero to one methylated markers in the *HIST1H4F*, *PCDHGB6*, *NPBWR1*, *ALX1* and *HOXA9* genes were characterized by a longer relapse-free survival time than those with two or more hypermethylated markers; 48% from the enriched methylated group relapsed, as compared with only 18% of those in the less methylated group [94].

### 3.2. Deamination of Cytosine Bases

#### 3.2.1. Spontaneous Deamination

**Figure 3:**
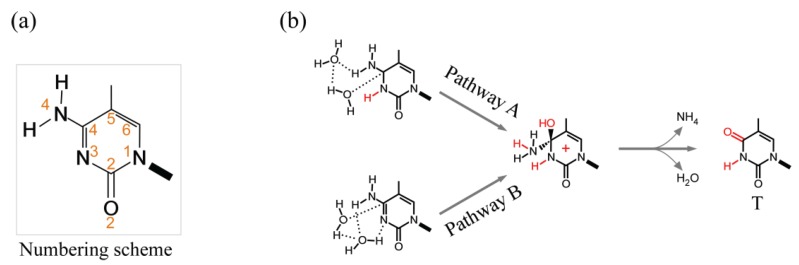
(**a**) Numbering scheme for cytosines; (**b**) Pathways for the spontaneous deamination of C and 5mC (only 5mC is shown).

The rate constants for the spontaneous deamination of cytosine (C) and protonated C to uracil (U), 5mC to T, 5hmC to 5hmU, 5fC to 5fU and 5caC to 5caU (Figure 1, bottom) have been determined from both the extrapolations of Arrhenius plots and genetic assays [95,96,97,98,99,100]. In double-stranded DNA, deamination rates for C and 5mC are extremely slow, of the order of 10^−13^ s^−1^ (Table 2), which translates into half-lives ranging between ~30,000 to ~85,000 years. Rates increase approximately 3-orders of magnitude in both single-stranded DNA and in isolated deoxyribonucleotides, supporting the view that hydrogen bonding plays a major role in shielding cytosines from spontaneous deamination. Comparison of the data given in Table 2 indicates that rates of deamination for 5mC are only marginally higher than for cytosine. Rates increase by an additional 3-orders of magnitude upon protonation; however, since cytosine protonation occurs at acidic pH (pKa = 2.4) within a C:G Watson-Crick base-pair [101], it is unlikely to play a significant role *in vivo*. That DNA melting is rate-limiting for cytosine deamination is further suggested by evolutionary studies, which indicate that CG:CGs embedded within G+C-rich areas (H isochores), and thus characterized by increased melting temperatures, have been depleted to a lesser extent than CG:CGs embedded in G+C-poor (L isochores) regions, which melt at lower temperatures [102]. Further support is provided by the finding that C→T transitions decrease gradually with increasing nucleosome occupancy score in comparative studies of *S. cerevisiae*, medaka (*Oryzias lapites*) and *C. elegans* genomes [103]. Analysis of the hydrolytic deamination reaction using density functional theory shed considerable light on the requirements for base unpairing and the effect of protonation [104]. Two pathways have been identified (Figure 3). In pathway A, upon protonation of N3 (Figure 3a), nucleophilic addition of a first water molecule to carbon C4, leads to the formation of a tetrahedral intermediate with the assistance of a second water molecule (Figure 3b). The C4-N4 bond is then broken and a proton transfer takes place from the hydroxyl group at C4 to NH_3_, thereby forming thymine and an ammonium cation. In pathway B, nucleophilic addition of the first water molecule to C4 occurs on the neutral 5mC (Figure 3b), again with the assistance of a second water molecule, yielding a neutral tetrahedral intermediate (not shown). The exocyclic amino group is then protonated through an intermolecular proton transfer, after which the reaction proceeds as in pathway A. For both pathways, the nucleophilic addition is the rate-determining step; however, whereas nucleophilic addition to carbon C4 of 5mC is easier than to the N3-protonated form, the trend is reversed in the case of C and N3-protonated C. Thus, deamination of 5mC is more difficult than that of C in pathway A, whereas the opposite is seen in pathway B.

This study has several implications. First, nucleophilic attack and formation of the tetrahedral intermediate cannot occur on duplex DNA; second, only pathway B is compatible with the greater susceptibility of 5mC to deamination, compared with C; third, a protonated base is not required for pathway B; and fourth, for pathway B, the activation free energy for 5mC (134.1 kJ/mol in aqueous solution) is only 4.4 kJ/mol less than that associated with C (138.5 kJ/mol in aqueous solution), implying that the susceptibility to deamination of 5mC relative to C is no more than 4-5-fold. In summary, these investigations show that (1) spontaneous deamination of cytosine bases only occurs in single-stranded DNA; (2) deamination of 5mC is only marginally more efficient than for C; and (3) all C5 substituted bases, with the exception of 5caC, display detectable (and rather similar) rates of deamination.

#### 3.2.2. ROS-Induced Deamination

ROS, such as the non-radical hydrogen peroxide (H_2_O_2_) and free superoxide radicals (O_2_^−^), are generated as a result of mitochondrial respiration, from the activation of growth factor receptors through NADPH oxidase, the arachidonic acid cascade and others, and play crucial roles as signal transduction molecules and neuroregulators [105,106]. H_2_O_2_ may also generate free radicals, including the hydroxyl radical (OH), the most potent oxidizing radical generated by the cell, through routes including ionizing radiation, interactions with O_2_^−^ through the Haber-Weiss reaction and by interactions with transition metal ions [Fe(II) and Cu(I)/Cu(II)] through Fenton chemistry, as exemplified in reaction 1.

Cu(II) + H_2_O_2_ → Cu(I) + H_2_O + H^+^; Cu(I) + H_2_O_2_ → Cu(II) + OH + OH^−^ (1)

Free radicals constitute an important endogenous source of damage to DNA and other molecules, and therefore strict homeostatic controls exist in the cell between the generation and neutralization of ROS species by catalase, superoxide dismutase 1 and 2 (SOD1 and 2), the glutathione peroxidase (GPX) and peroxiredoxin (PRX) families of detoxifying enzymes and other antioxidants, such as vitamins C and E [106,107]. A number of studies in leukemia, breast cancer, ovarian cancer, benign and malignant prostate cancer, non-small cell lung carcinoma, cervical squamous cell carcinoma, stomach cancer, and Hodgkin’s disease patients all concur with the conclusion that levels of ROS detoxifying enzymes are generally lower in cancer than in surrounding normal tissue, leading to oxidative stress, *i.e.*, altered ROS homeostasis in favor of increased steady-state levels of ROS [107]. In addition, as in the case of *BCR-ABL1* translocations in myeloid leukemia, Rac2 activation has been shown to reduce the mitochondrial membrane potential (Δ*Ψ_m_*), thereby inducing electron leakage from the mitochondrial respiratory chain complexes I-III and II-III (MRC-cIII) and, as a consequence, a 2- to 6-fold increase in cellular ROS [108].

Treatment of duplex oligonucleotides containing methylated and unmethylated CG:CGs with Cu(II)/H_2_O_2_/ascorbate to effect Fenton-type reactions led to much more frequent modifications of 5mC than C [109]. One of the main products involved the saturation of the C5-C6 double-bond of 5mC, to yield 5-methyl-5,6-dihydroxy-5,6-dihydro-2'-deoxycytidine (5-methyl-2'-deoxycytidine glycol, 5mCg). Kinetic determinations of the spontaneous deamination of two stereoisomers of 5mCg, *i.e.*, 5mCg(*5S*,*6S*) and 5mCg(*5R*,*6R*) in duplex DNA, which affords 5,6-dihydroxy-5,6-dihydrothymidine (thymidine glycol, Tg) [110], indicated rates in the range of ~10^−6^ s^−1^, similar to the values determined for isolated nucleotides. Thus, ROS-induced saturation of the C5-C6 double-bond in 5mC increases rates of deamination by ~4 orders of magnitude with respect to single-stranded DNA, and ~7 orders of magnitude with respect to unmodified 5mC in double-stranded DNA. The susceptibility of duplex DNA to damage by Fenton-type reactions has been assessed using 5S rDNA, either alone or upon reconstitution on nucleosome particles. The number of single base lesions was found to be 8-fold higher on nucleosomal DNA than on isolated DNA, implying that Fenton chemistry is not only unrestricted by chromatin compaction but actually appears to be facilitated [111]. Although the roles of histone octamers in permitting DNA damage are not fully understood, X-ray crystal structure studies revealed the presence of many divalent metal binding sites in nucleosome particles [112], and several peptide models of histones H2A, H2B, H3 and H4 have been shown to coordinate Cu(II), mostly through macrochelate rings involving histidine and carboxylate groups [113]. The extremely short (<1 ns) half-life of OH likely restricts Fenton chemistry at sites of OH generation. Thus, given that H_2_O_2_ is relatively stable and able to diffuse across cells, copper coordination within nucleosome particles might provide a suitable environment for oxidative DNA damage in chromatin. Determinations of copper concentrations by both atomic absorption spectroscopy and X-ray fluorescence in plasma and tumor samples from several types of cancer indicate that levels are usually higher (up to 2- to 3-fold) in cancer patients than in normal controls [107]. Indeed, high levels of copper appear to be required for tumor growth [114].

**Table 2:** Rates of spontaneous deamination for cytosines.

Deaminating base	Sequence context	Deaminated base product	Deamination rate at 37 °C (*s*^−1^)	References
C	Free nucleoside	U	9.4 ± 0.5 × 10^−10^	[95]
3mC^+^	Free nucleoside	3mU	5 × 10^−7^	[100]
3mC^+^	Free nucleotide	3mU	13 × 10^−7^	[100]
5mC	Free nucleoside	T	7.8 ± 0.3 × 10^−10^	[95]
5hmC	Free nucleoside	5hmU	5.8 ± 0.8 × 10^−10^	[95]
5fC	Free nucleoside	5fU	1.2 ± 0.2 × 10^−9^	[95]
5caC	Free nucleoside	5caU	not detected	[95]
5mCg(*5S*,*6S*)	Free nucleoside	Tg	1.1 × 10^−5^	[115]
5mCg(*5R*,*6R*)	Free nucleoside	Tg	8.6 × 10^−6^	[115]
C	ssDNA	U	2.1 × 10^−10^	[96]
C	ssDNA	U	~1 × 10^−10^	[98]
5mC	ssDNA	T	9.5 × 10^−10^	[96]
C	dsDNA	U	2.6 × 10^−13^	[97]
C	dsDNA	U	4 × 10^−13^	[99]
C	dsDNA	U	~7 × 10^−13^	[98]
5mC	dsDNA	T	5.8 × 10^−13^	[97]
5mC	dsDNA	T	1.5 × 10^−11^	[99]
5mCg(*5S*,*6S*)	dsDNA	Tg	5.2 × 10^−6^	[109]
5mCg(*5R*,*6R*)	dsDNA	Tg	7.0 × 10^−6^	[109]

C, cytosine; 5mC, 5-methylcytosine; 3mC^+^, *N*^3^-methylcytosine; 5hmC, 5-hydroxymethylcytosine; 5fC, 5-formylcytosine; 5caC, 5-carboxycytosine; 5mCg(*5S,6S*) and 5mCg(*5R,6R*), 5-methylcytosine glycol stereoisomers; U, uracil; 3mU, *N*^3^-methyluracil; T, thymine; 5hmU, 5-hydroxymethyluracil; 5fU, 5-formyluracil; Tg, thymine glycols.

In summary, oxidation of 5mC by copper ions and ROS generate cytosine glycol intermediates, which deaminate at high rates to yield Tg:G mispairs. DNA oxidation by Fenton chemistry is enhanced in nucleosomal DNA, in which several coordination sites for copper and other metal ions have been identified. Both ROS and copper concentrations have been found to be enhanced in tumors, raising the possibility that ROS-dependent deamination at methylated CG:CG sites may contribute to mutation in cancer.

#### 3.2.3. Enzymatic Deamination by Single-Stranded DNA-Specific AID/APOBECs

AID/APOBEC enzymes catalyze the deamination of cytosine to uracil on single-stranded DNA (Figure 1, bottom). The active site of APOBEC enzymes is recognized by a conserved motif (H-X-E-X_23–28_-P-C-X-C), in which a coordinated zinc ion carries out the nucleophilic attack during the deamination reaction. In the high-resolution crystal structures of human APOBEC2 and the catalytic domain of APOBEC3G (aa 197–380), a water molecule serves as a hydrogen donor, whereas a conserved glutamate residue (E100 in APOBEC2 and E259 in APOBEC3G) functions as a proton shuffler during the hydrolytic cycle [116]. As with other DNA metabolizing enzymes, the target cytosine is flipped-out and inserted into the active site; because the flipping step appears to involve passive DNA breathing, it probably accounts for the greater enzymatic activities observed for deamination of single-stranded, as opposed to double-stranded, nucleic acids [117,118]. Whether AID/APOBEC enzymes deaminate cytosines modified at C5, including 5mC, 5hmC, 5fC and 5caC has been controversial, with recent studies reporting generally weak or no activities towards C5-substituted cytosines [118,119,120], and earlier work reporting strong activities on 5mC by human AID and rat APOBEC1 [121]. Experiments performed by scoring mutations either in viral DNA or *in vitro* with model sequences indicate strong effects on deamination rates by nucleotides flanking the target cytosine: optimal substrates include (C/G)TC(A/G) for APOBEC3B [122,123], T(T/C)C for APOBEC3C [124], CCCA for APOBEC3G [122,124,125,126,127], TTCT for APOBEC3F [124,125,126], WRCY (W = A or T; R = A or G; Y = C or T) for AID [128], although comparative analyses using enzymatic kinetic constants awaits further work. In summary, APOBEC enzymes favor deamination of unmodified cytosine residues, to yield C-to-U modifications, in single-stranded DNA and in a sequence-dependent manner that is specific to each family member.

### 3.3. Sequence Context-Dependent Guanine Oxidation Products

#### 3.3.1. Guanine

DNA oxidation plays a significant role in the pathophysiology of cancer, with epidemiological studies demonstrating a strong association between the generation of ROS and reactive nitrogen species (RNS) from chronic inflammation and increased cancer risk [129]. Guanine has the lowest redox potential of all DNA bases, and it has consistently been found to be a highly susceptible site for reactions with a variety of agents, including singlet oxygen, OH radicals, peroxynitrite, UV radiation with riboflavin and many others [130]. For example, in a study in which duplex DNA was subjected to 266 nm wavelength laser pulses as a source of photonic ionization, the quantum yield for the formation of 8-oxoG was much higher than that of oxidized nucleosides arising from the degradation of the other bases [131]. In addition, extensive experimental evidence supports a role for sequence context in terms of the chemistry and extent of DNA damage at guanine residues. Pioneering work in which DNA cleavage was induced by riboflavin as an electron-accepting photosensitizer in double-stranded 30-mers containing a target G in different sequence contexts (5'-TXGYT-3'), showed that the extent of cleavage at the target G depended upon DNA flanking sequence composition [73], implying that the ease of losing an electron by the target G was also dependent on flanking sequence. Indeed, computations of the ionization potentials (*i.e.*, the energy required to abstract an electron) (IPs) for the target G were found not only to differ with varying X and Y in a 5'-XGY-3' context, but also to correlate inversely with the extent of cleavage [73]. Specifically, the ionization potentials at a G followed by a 3' A or G were found to be up to 0.44 eV lower than when followed by a 3' T or C, whereas the base composition 5' of a G made little (<0.1 eV) contribution [74]. Hence, these and other investigations have together laid the foundation for the concept that a positive charge (a hole) inserted into DNA by abstracting an electron migrates through base stacking, either by hopping from one base to the next over long distances or by a tunneling mechanism over short distances (1–3 bases) [132], from the original location to sites of lowest IPs, *i.e.*, 5' G in GA and GG sequences.

#### 3.3.2. 8-oxoG

**Figure 4:**
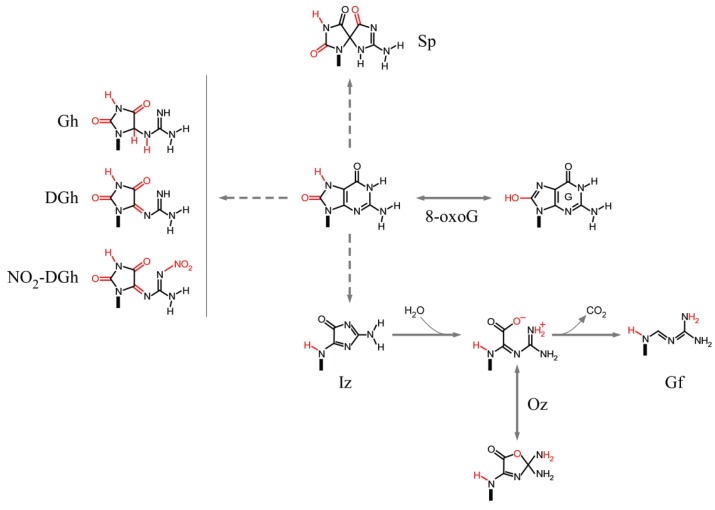
Sequence context-dependent reaction products of 8-oxoG.

In addition to unmodified bases, sequence context-dependent reactivity has also been observed for 8-oxoG, one of the main products of DNA exposed to ROS and RNS. 8-oxoG is several orders-of-magnitude more susceptible to further oxidation than G itself due to a lower ionization potential (6.93 *versus* 7.31 eV for unstacked 8-oxoG and G, respectively) [133], yielding more stable secondary oxidation products, including dehydroguanidinohydantoin (DGh), *N*-nitro-dehydroguanidinohydantoin (NO_2_-DGh), 5-guanidinohydantoin (Gh), 2-imino-5,5'-spirodihydantoin (Sp), 2,5-diamino-4*H*-imidazol-4-one (imidazolone, Iz), its hydrolysis product 2,2,4-triamino-5(2*H*)-oxazolone (oxazolone, Oz) [134] and guanidinoformimine (Gf), the decarboxylated product of Oz [135] (Figure 4).

Earlier studies demonstrated that 8-oxoG reactivity to a variety of oxidants, including NiCR/KHSO_5_, IrCl_6_^2−^, IrBr_6_^2−^, Fe(CN)_6_^3−^, SO_4_^−^ and ^1^O_2_, increased when located 5' to a G (8-oxoGG) compared to 3' to a G (G8-oxoG) [136], a trend that followed the computed sequence-dependent ionization potentials (6.38 eV for 8-oxoGG and 6.51 eV for G8-oxoG) [133]. More recent studies further established the sequence-dependent reactivity of 8-oxoG in duplex oligonucleotides to UVA-irradiated riboflavin to follow: C8-oxoGA ≈ A8-oxoGG > G8-oxoGG > C8-oxoGT > T8-oxoGC > A8-oxoGC, supporting a model whereby indiscriminate removal of electrons from all four nucleobases by riboflavin creates holes that migrate to sites of lower IPs (8-oxoG), with 8-oxoG reactivity modulated by sequence-dependent variations in the IPs by neighboring bases [137]. In addition to the extent of reactivity, also the types of products formed by riboflavin-oxidized 8-oxoG varied with flanking sequence composition. For example, although three main products were generally observed (Sp > Gh > Iz), at low riboflavin concentrations (<15 μM) oligonucleotides containing G8-oxoGG, C8-oxoGT and T8-oxoGC yielded relatively high levels of Sp that decreased as a function of increasing riboflavin concentration. By contrast, at riboflavin doses >30 μM, DGh was the most abundant species in some sequence contexts (G8-oxoGG and C8-oxoGA), with Iz matching DGh in the A8-oxoGG and G8-oxoGG sequence contexts. In contrast to riboflavin, nitrosoperoxycarbonate (ONOOCO_2_^−^), generated from macrophage-derived nitric oxide (NO) and superoxide (O_2_^−^), failed to yield sequence-dependent 8-oxoG reactivity and displayed a rather uniform spectrum of oxidation products, which were dominated by DGh > Oxaluric acid > NO_2_-DGh [138]. Likewise, Gh and Sp have been established as the main products of G and 8-oxoG oxidation by peroxynitrite, peroxyl radicals, and hypochlorous acid, reactive species also released by macrophages during an inflammatory response [139]. Thus, as noted by Lim *et al.* [138], “the observation of strong sequence context effects on the final chemistry of DNA oxidation complicates our understanding of the mechanistic basis for both mutation frequency and mutational spectra caused by DNA damage *in vivo*”, a task that is further complicated by sequence-dependent variations in the rates of DNA repair of individual DNA lesions. The analyses reported above clearly point to a critical role being played by charge (electron) transfer in the sequence context-dependent oxidation of DNA and the migration of the original sites of damage to distant sites of lower IPs, mostly G and 8-oxoG in the GA, GG, 8-oxoGA, and 8-oxoGG contexts.

#### 3.3.3. Charge Transfer in Nucleosomal DNA

Whereas bases in single-stranded DNA are generally more easily oxidized than in double-stranded DNA [138], the stability of stacking interactions in duplex DNA enables the charge transfer process towards guanine bases to take place more efficiently in duplex DNA than in single-stranded DNA. For example, increasing the ionic strength, which results in a more stable duplex, also enhanced the yield of 8-oxoG and, concomitantly, decreased the yield of thymine and adenine oxidation products upon 266 nm laser pulses in isolated DNA [131]. Charge transfer was also shown to occur more efficiently in chromatinized DNA than in naked DNA. A detailed study of the location and types of guanine oxidation products generated by UVA photodamage along a duplex DNA fragment wrapped around a nucleosome core particle (NCP) indicated that, whereas in naked DNA lesions were mostly localized at the distal sites, in nucleosomal DNA there was substantial enhancement of internally (*i.e.*, in contact with the NCP) damaged guanine sites [140]. Surprisingly, removing the histone tails from nucleosomes, most of which were in molecular contact with the packaged DNA, was sufficient to abrogate the effects of nucleosomal packing on long-range charge transfer, implying that weakening histone-DNA interactions also dampened the efficiency of charge transfer along DNA. In addition, a shift in the nature of guanine lesions was observed, from Oz mostly in the linker regions and in naked DNA to 8-oxoG for those sites in closest contact with the NCP. Because the guanine radical cation (G^+^), a key intermediate in guanine oxidation, may react with either oxygen or water to yield Oz and 8-oxoG, respectively, these results clearly show that the NCP shields G^+^ from reacting with molecular oxygen. As the authors pointed out, the implications of this study are two-fold: first, “the enhancement of damage in the most tightly packaged nucleosomes could result in enhanced guanine oxidation in heterochromatin *versus* euchromatin”; and second “the distribution of guanine oxidation products is modulated by nucleosomal packaging. Therefore, the spectrum of guanine lesions generated by DNA oxidation could vary in different regions of chromatin”.

In summary, sites of oxidation in DNA may migrate from their original location to sites of lower ionization potentials, e.g., predominantly G in the context of GA and GG sequences (charge/electron transfer); charge transfer also occurs towards 8-oxoG, which yields different oxidation products depending on the nature of the oxidizing agent and flanking sequence composition. Finally, charge transfer in DNA is favored in chromatin, where guanine oxidation products are modulated by their position along the NCP.

## 4. DNA Repair Pathways and Synthesis across Modified Bases and Mismatches

### 4.1. Base Excision Repair

Base excision repair (BER) is initiated by the activity of a DNA glycosylase that recognizes small perturbations in the DNA helical structure caused by base modifications or a mismatched base-pair [141]. The basic steps of BER, the distinction between short and long patch repair, the nature of monofunctional *versus* bifunctional enzymes and the involvement of Pol β in cancer have been thoroughly reviewed [141,142,143]. Herein, we shall focus on those BER enzymes that have been shown to process the modified bases and mismatches described above.

#### 4.1.1. Modified CG:CG Sites

Two monofunctional DNA glycosylases display a preference for correcting T:G and U:G mismatches in the CG:CG sequence context in double-stranded DNA, methyl-CpG binding domain protein 4 (MBD4) and thymine DNA glycosylase (TDG). MBD4 contains an N-terminal methyl-CpG binding domain and a C-terminal DNA glycosylase domain that acts on T:G, 5hmU:G and U:G mismatches with relative rate constants of 0.5, 1.0 and 1.7 min^−1^, respectively [144], and on Tg:G with half the efficiency observed for T:G. Thus, the enzyme is poised to recognize deamination products of 5mC:G, 5hmC:G and C:G within CG:CG sequences [145]. Consistent with these activities *in vitro*, *Mbd4*^−/−^ mice display a ~3-fold increase in C→T transitions at CG:CG sites relative to wild-type littermates [146,147]; however, a direct role for *Mbd4*^−/−^ in accelerating tumorigenesis has not been confirmed [148]. Competition experiments indicate that CG:CG methylation enhances MBD4 binding and that, whereas glycosylase activity is observed on reconstituted chromatin, activity is enhanced upon histone tail acetylation, consistent with increased accessibility of the target sites on a less compact chromatin environment [149]. TDG actively processes a number of lesions resulting from oxidation, alkylation and deamination of C, 5mC, 5hmC, T and A, with the strongest activities observed on U:G > T:G > Tg:G mismatches. The enzyme also cleaves the products of 5hmC oxidation, 5fC and 5caC. The *TDG* gene is expressed at high levels in the G2-M and G1 phases of the cell cycle, and then rapidly declines at the onset of S-phase. Loss of *TDG* expression is embryonic lethal in mice and, indeed, a number of investigations support a role for TDG in demethylation during embyogenesis, whereas interactions with transcription factors, transcriptional coregulators, DNMT3a, DNMT3b and others, suggest a scenario in which coordinated CG:CG methylation/demethylation and chromatin organization serve to regulate gene expression [145]. A striking preponderance (86%) of C→T transitions at mutated CG:CG sites, which are normally methylated, was recently reported in a mismatch-repair (*PMS2*) deficient 13-year-old colorectal cancer patient with a heterozygous germline missense mutation in *TDG* [150], in line with a potential role for TDG in repairing C5-substituted C deaminated products at CG:CG sites.

**Figure 5:**
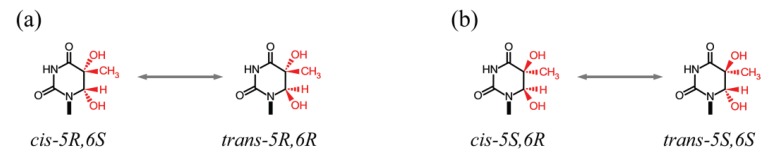
(**a**) *cis-trans* stereoisomer pair of *5R* thymine glycol; (**b**) *cis-trans* stereoisomer pair of *5S* thymine glycol.

Because thymidine glycol may exist in four different configurations, *5R_cis/trans_* (Figure 5a) and *5S_cis/trans_* (Figure 5b) pairs, the efficiency of repair by DNA glycosylases will vary depending upon whether Tg isomers oppose G (Tg:G), which results from 5mC oxidation and deamination, or A (Tg:A), which results from T oxidation. Under conditions of single turnover, the stereoselectivity of nth endonuclease III-like 1 (NTHL1, a bifunctional enzyme with β-lyase activity) was similar for Tg(*5R*):A and Tg(*5R*):G, but the amount of Tg(*5R*) cleaved was ~13-fold higher than for the Tg(*5S*) due to stronger product inhibition by the latter. By contrast, for nei endonuclease VIII-like 1 (NEIL1, a bifunctional enzyme with β,δ-lyase activity), no stereoselectivity was detected; however, Tg:G was excised much more rapidly than Tg:A, suggesting that NEIL1 may be primarily involved in the repair of modified CG:CG sites [151]. *Nth1*^−/−^*Neil1*^−/−^ double mutant, but not single mutant, mice developed a high incidence of lung and liver tumors after the first year, implying overlapping roles in DNA repair [152].

NEIL1 synthesis is activated during S-phase, and NEIL1 has been proposed to act at the replication fork to remove oxidative DNA lesions in a scheme involving: (1) damage recognition on the single-stranded template but cleavage inhibition by replication protein A (RPA); (2) fork reversal, which places the lesion back into duplex DNA; (3) base cleavage; and (4) resumption of the collapsed replication fork and DNA synthesis. Although NEIL2, which displays substrate specificities similar to NEIL1, was able to partially complement NEIL1 at the replication fork, its activity is believed to be more relevant during transcription [153]. A study on promoter methylation for 160 DNA repair genes in ~40 head and neck squamous cell carcinoma samples and controls identified *NEIL1* as the most prominently hypermethylated gene, with 81% samples (35/43) displaying significant hypermethylation relative to controls, suggesting a role for diminished DNA repair activity in cancer onset or progression [154].

#### 4.1.2. Uracil

Two additional monofunctional DNA glycosylases, uracil-DNA glycosylase 2 (UNG2) and single-stranded selective monofunctional uracil-DNA glycosylase 1 (SMUG1), serve to remove uracil from nuclear DNA [141]. UNG2 is a single-stranded DNA-specific enzyme that plays an indispensable role in somatic hypermutation (SHM), which is part of the antigen-driven high-affinity antibody diversification program in follicular B cells, by removing uracil generated by AID at WRCY sequence hotspots. *In vitro*, SMUG1 is active either on single-stranded or double-stranded DNA, depending on salt concentrations. However, at physiological mono and divalent metal ion concentrations, SMUG1 is active only on double-stranded DNA, and therefore it is considered to be a double-stranded-specific DNA glycosylase. This distinction is crucial, since it places UNG2 as the sole enzyme acting on uracil in single-stranded DNA *in vivo*. Thus, in addition to sequence-specificity, the ability of RPA, a single-stranded DNA binding protein, to recruit UNG2 to single-stranded DNA has been proposed as a key feature that restricts UNG2 (rather than SMUG1, TDG or MBD4) activity to SHM [155]. In mice, during SHM, UNG2-generated AP sites are “copied” by error-prone translesion synthesis (TLS) polymerases during DNA replication, including Rev1 and Pol η, yielding C→T, C→G and C→A mutations [128]. In chronic myeloid leukemia in chronic phase (CML-CP) hematopoietic stem cells, the kinase activity associated with the *BCL-ABL1* translocation was found to inhibit UNG2 activity, thereby promoting mutations arising from increased ROS-mediated oxidative base lesions [156].

#### 4.1.3. Guanine Lesions

Two bifunctional double-strand-specific DNA glycosylases, 8-oxoguanine DNA glycosylase 1 (OGG1) and mutY homologue (MUTYH), act upon 8-oxoG, a highly miscoding lesion that instructs A incorporation (8-oxoG:A base-pairs) by the replicative DNA Pol δ/ε. Κnockout mice for both enzymes are strongly prone to lung, ovarian cancers and lymphomas, and have shortened life spans [157], whereas human germline biallelic *MUTYH* mutations have been implicated in MUTYH-associated polyposis, a condition associated with increased risk of colorectal cancer [158]. OGG1 cleaves 8-oxoG only when paired with C, owing to specific contacts made with both bases, which trigger catalysis [159]. By contrast, MUTYH specifically cleaves the A base in 8-oxoG:A base-pairs by using a central interconnector domain (ICD) to coordinate the action between the N-terminal catalytic domain and the C-terminal 8-oxoG recognition domain. In addition, the ICD serves as a structural scaffold to direct MUTYH activity to replication foci through specific interactions, including PCNA and Rad9-Rad1-Hus1 (the 9-1-1 complex) [160]. After induction of oxidative stress, the co-localization of OGG1-containing BER patches with H3meK4 or acetylated histone H4 in euchromatic regions and the exclusion from heterochromatic regions suggests that chromatin compaction hinders BER [161]. These conclusions are in line with dinucleosome reconstitution experiments *in vitro*, in which 8-oxoG cleavage in the linker region separating the two nucleosomes was unhindered in the absence of H1, but was decreased ~10-fold upon H1 binding to the linker [162].

*In vitro*, Oz in Oz:C and Gh in Gh:C mispairs in duplex oligonucleotides were cleaved by NEIL1 as efficiently as the well-recognized pyrimidine lesion 5hU:G, implying that guanine oxidation lesions, in addition to pyrimidine lesions, are good substrates for the enzyme. Similar results were obtained with NTHL1, although Gh was cleaved slightly more efficiently than Oz [163]. NEIL1 and NHTL1 also cleaved Oz:G as efficiently as Oz:C, which would give rise to G→C transversions; Oz:G mispairs are predicted to arise from Pol α or ε replication across Oz, as mentioned below.

Although the ability of NEIL1 and NEIL2 to remove Sp and Gh lesions has been well documented [164,165,166], a critical role for NEIL3 has recently emerged [167]. *Neil3*^−/−^ mice exhibited learning and memory deficits, impaired proliferation of neural stem/progenitor cells [168], and tissue extracts from *Neil3*^−/−^ mice, but not from wild-type littermates, displayed defective nicking activities on hydantoin lesions only when present on single-stranded DNA [169]. The purified human catalytic domain of NEIL3 was also found to display strong preference for Sp and Gh when compared to several other lesions, including 5-OHC and 5-OHU, with the greatest turnover number (0.035 s^−1^) on Gh in single-stranded DNA, as assessed from single turnover experiments, ~2-fold higher than on double-stranded Gh and single-/double-stranded Sp. Thus, removal of Sp and Gh lesions appears to depend critically on NEIL3. Interestingly, the enzyme elicited uncoordinated cleavage and β-lyase activities, suggesting that it can act both as a monofunctional and as a bifunctional glycosylase [170].

### 4.2. Lesion Bypass

**Figure 6:**
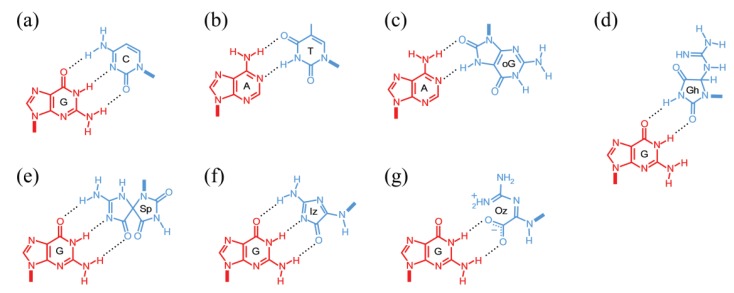
(**a**) Canonical G:C base-pair; (**b**) Canonical A:T base-pair; (**c**) 8-oxoG(*syn*):A(*anti*) base-pair; (**d**) Gh:G base-pair; (**e**) Sp:G base-pair; (**f**) Iz:G base-pair; (**g**) Oz:G base-pair. For noncanonical base-pairs, the templating base is shown in blue.

Base lesions that remain unrepaired can serve as templates during DNA synthesis, and are either copied by the normal replicative DNA polymerases (Pol α, δ and ε) or alternatively may block replication, in which case they are bypassed by one of 10 specialized polymerases lacking 3'→5' proofreading exonuclease activity during TLS, which limits DNA synthesis to a few bases across the blocking lesion [43,142]. Of the modified bases described above, C5 modified cytosines direct mostly guanine incorporation, and hence are not mutagenic; by contrast, their deaminated counterparts (thymine and uracil derivatives) enable the incorporation of adenine by replicative polymerases [171], which yields C→T (G→A) transitions. Tg stereroisomers block replication; investigations *in vitro* with DNA Polymerase gp43 from bacteriophage RB69 (a polymerase of the B-family, which in humans includes Pol α, δ, ε and ζ) revealed that whereas Tg is weakly bypassed and correctly paired with A, extension is inhibited by the enzymatic exonuclease activity [172] and, thus, extended through TLS by Pol α, ζ, ν, η or θ, either alone or in combination [43,173,174,175,176,177,178,179]. Thus, TLS across Tg:G base-pairs originating from 5mC oxidation and subsequent deamination are expected to lead almost quantitatively to C→T (G→A) transitions. 8-oxoG is able to functionally mimic thymine in the *syn* conformation, and DNA synthesis by replicative polymerases has been shown to yield both the correct 8-oxoG:C base-pair and the incorrect 8-oxoG(*syn*):A(*anti*) Hoogsteen base-pair [180,181,182] (Figure 6a–c) in ~3:2 ratios and to be extended, mostly by Pol λ [182,183,184,185,186,187]. The 8-oxoG:A base-pair would therefore give rise to G→T (C→A) transversions.

The crystal structure of bacteriophage B-family Polymerase RB69 bound to the templating *R*-stereoisomer of Gh revealed that the base was flipped in a non-templating position. However, the results also suggested that either slow rotation by the *R*-isomer or, more effectively, the *S*-isomer would present the pyrimidine-like hydantoin side to the enzyme, thereby instructing incorporation of a purine (A or G) [188]. Indeed, a Y567A mutant of RB69 was found to insert both bases with >100-fold increased efficiency, whereas extension was blocked [189]. These data suggest that, as in the case with Tg stereoisomers, Gh:A and Gh:G are switched to the enzymatic exonucleolytic domain, thereby triggering a futile incorporation/degradation cycle that effectively blocks DNA replication and renders Gh an obligate mutagen. Thus, a TLS extender polymerase may assist in lesion bypass across Gh *in vivo*; in addition, Pol η was found to bypass Gh efficiently and to incorporate either A or G in primer extension assays [190]. Although Sp was also found to yield G→C and G→T transversions [191], no structural data are currently available for polymerease:Sp complexes.

In primer extension assays, Pol η was found to partially extend past the Oz, Iz and Gf lesions (Oz > Iz > Gf), whereas Pol κ was almost completely blocked [135]. Sequence analyses of the extended products indicated that Pol η incorporated either C (55%–65%) or G (35%–45%), whereas Pol κ incorporated C (41%–58%), G (25%–37%) and A (16%–33%), across all lesions. Thus, T incorporation seems to be limited with both TLS polymerases. How these lesions are processed by replicative DNA polymerases and the extent to which each leads to SBSs remains to be determined. The stabilization energies (*ΔE*) for a number of isomers of the oxidative guanine products Gh, Sp, Iz and Oz base-paired with G (Figure 6d–g) have been computed by *ab initio* molecular orbital calculations; using density functional theory, *ΔE* values varied from 28.2 kcal/mol for Sp:G to 20.7 kcal/mol for Oz:G (30.9 kcal/mol for the canonical G:C base-pair) [130,192].

### 4.3. Transcription-Coupled Repair

Given that cancer genome analyses have indicated that the number of SBSs is often lower at sites of putative base lesions on the transcribed strand than on the non-transcribed strand [13,30,193], we will briefly discuss some key aspects of transcription-coupled repair. Nucleotide excision repair (NER) is considered to be the main pathway for the repair of UV photo-induced DNA lesions; CPDs and 6-4 pyrimidine-pyrimidone photo products (6-4PP) [194], and defects in NER components are associated with inherited DNA repair syndromes such as xeroderma pigmentosum [195] and Cockayne syndrome [194], that display severe hypersensitivity to sunlight. NER also actively repairs many types of DNA adducts that cause helical distortions, including environmental mutagens such as benzo[*a*]pyrene and other PAHs, aromatic amines, oxidative endogenous lesions such as cyclopurines, and adducts caused by cancer chemotherapeutic agents, including cisplatin [196]. NER comprises two distinctive subpathways, global genome NER (GG-NER) and transcription-coupled NER (TC-NER), which acts specifically on the transcribed strand of actively transcribed genes [194]. TC-NER is activated by the physical blockage imposed by bulky DNA adducts and abasic sites on RNA Polymerase II (RNAPII), but not by small lesions such as thymine glycols and 8-oxoG [197,198]. RNAPII is found in transient association with Cockayne syndrome type B protein (CSB, the product of the *ERCC6* gene) and the UV-stimulated scaffold protein A (UVSSA)–ubiquitin-specific peptidase 7 (USP7) complex. RNAPII stalling is believed to stabilize RNAPII/CSB interactions and activate the UVSSA/USP7 complex, thus protecting CSB from degradation through deubiquitination. Stabilized CSB recruits a complex that includes Cockayne syndrome WD repeat protein (CSA, product of the *ERCC8* gene), damage-specific DNA binding protein 1 (DDB1), cullin 4A (CUL4A) and others, which mediates downstream events, including chromatin remodeling, permitting backtracking of the RNAPII complex and exposing the lesion for repair by the GG-NER complex. Specific DNA adducts have been shown to be repaired exclusively by TC-NER but not by GG-NER. For example, exome sequencing of urothelial carcinomas of the upper urinary tract associated with chronic exposure to aristolochic acid, a natural compound from traditional herb medicine, revealed a characteristic A→T (T→A) mutational signature on non-transcribed strands leading to splicing defects [199,200], which was attributed to a failure of GG-NER, but not TC-NER, to recognize aristolactam-DNA adducts [201]. In addition to its role in TC-NER, CSB has been reported to facilitate lesion bypass by the RNAPII complex [202], to associate with components of BER, including OGG1, NEIL1 and AP1, and to elicit critical functions in mitochondria related to DNA repair, ROS homeostasis and others [203,204,205].

More recently, immunoprecipitation experiments in human gastric epithelial AGS cells revealed a direct association between the DNA glycosylase NEIL2, RNAPII and heterogeneous nuclear ribonucleoprotein U (hnRNP-U) [206]. On a 51-mer oligonucleotide, NEIL2 activity on a single 5-OHU lesion was stimulated 5- to 6-fold by hnRNP-U and likewise, on a plasmid system, a reconstituted transcription-repair complex comprising RNAPII, NEIL2, hnRNP-U and the BER components Pol β, Lig IIIa, PNK was proficient in repairing a 5-OHU lesion on the transcribed, but not on the non-transcribed, strand under conditions of active transcription. Co-localizations of NEIL2 with hnRNP-U with actively transcribed genes were confirmed by pulling down NEIL2-FLAG expressing AGS and neuroblastoma SK-N-BE2-(C) cells, followed by chromatin immunoprecipitation with hnRNP-U antibodies. Finally, a rise in mutations was inferred on selected, transcribed, genes in *NEIL2* knock-down compared to control cells. These composite data extend previous investigations [207], and document the preferential removal of oxidized DNA bases in actively transcribed genes by a system linking BER to the transcriptional apparatus. As already pointed out [206], it remains to be seen whether components of NER, such as CSB, also take part in BER related to transcription. In summary, whereas common oxidative DNA lesions may not be recognized by TC-NER, they are likely to represent a substrate for BER on the transcribed strand in association with transcription.

## 5. Proposed Mutational Mechanisms

Herein we have provided information on the most prominent SBS mutation patterns found in cancer genomes, *i.e.*, C→T transitions at CG:CG sites and substitutions at C:G base-pairs in the context of YC:GR dinucleotides, in an attempt to examine the validity of currently proposed models of mutagenesis. The main points may be summarized as follows: (i) higher fractions of SBSs have been found in cancer genomes within gene-poor regions, which are associated with heterochromatin and replicate late during the cell cycle, than in euchromatin, a pattern that mimics the one observed in population analyses; (ii) there are several established mechanisms for base substitutions at C residues, and their relative importance is only now being recognized. These involve deamination, oxidation, BER, TC-NER, TLS, and APOBEC activities; (iii) both guanine and 8-oxoG undergo sequence context- and ROS-dependent oxidation reactions that are consistent with an electron transfer (charge or hole migration) mechanism, whose efficiency is enhanced in the context of nucleosomal DNA; (iv) BER displays widely overlapping substrate specificity; however, NEIL enzymes appear to play a more prominent role in repairing guanine lesions, such as Gh and Sp, which are obligate mutagens during TLS; (v) most endogenous DNA lesions do not activate TC-NER; however, there are hints for a functional link between BER and transcription, leading to preferential repair on the transcribed strand.

C→T transitions at CG:CG sites have generally been attributed to faster spontaneous deamination of 5mC relative to C [13,16,17,19]. Our current analysis suggests that this explanation may be somewhat too restrictive. First, the fact that a family of C5-substituted Cs exists at CG:CG sites implies that C→T transitions are not limited to 5mC but, rather, to any of the C5-substituted species, *i.e.*, 5hmC, 5fC and 5caC. Second, as noted [208], the modest (5-fold at the most) increase in spontaneous deamination rates for C5-substituted Cs relative to Cs contrasts with the larger fractions of mutated Cs at CG:CG sites relative to non-CG:CG sites (up to 10–50 times), both in cancer and the germline [17,209]. Third, there does not seem to be a rational barrier to the possibility that 5mC oxidation and further deamination may yield Tg:G mismatches at CG:CG sites, which would then lead to C→T transitions during TLS. In fact, the observation that such a type of oxidation is facilitated by nucleosome occupancy raises the prospect for thymine glycols in Tg:G mispairs being a more prominent source of mutation at CG:CG sites than T:G mismatches, as previously pointed out [208]. The finding that C:G→T:A substitutions at CG:CG dinucleotides showed a strong positive correlation with the age at cancer diagnosis in ER– cancers, but not in ER+ cancers [193], further supports the conclusion that spontaneous deamination of 5mC may not be the only mechanism leading to mutations at CG:CG.

With respect to mutations at YC:GR sites, the gene expression data on APOBEC3B enzymes provide strong support for a role in cancer mutagenesis through U:G mismatch intermediates, as in the case of SHM. However, the extent to which the enzyme preference for single-stranded DNA may limit their activity genome-wide, possibly at sites of clustered and strand-coordinated mutations (kataegis) [13,210], remains to be determined. For example, whole-genome sequencing of gastric cancers, in which a prominent C→T signature at GC dinucleotides in coding-regions did not overlap with a preponderance of C→A transversions at CCT or TCA motifs genome-wide, were attributed to AID activation (on single-stranded DNA during transcription) and ROS/NOS, respectively, following *H. pylori* infection [56]. The findings that: (i) oxidative DNA damage occurs at YC:GR sites, which overlap with APOBEC3B specificity; (ii) numerous oxidation products can form at the target GR; (iii) efficient charge transfer and high mutation rates co-localize with heterochromatin; and (iv) some oxidation products of guanine are obligate mutagens during TLS, make it tempting to attribute a significant role for oxidative damage in mutations at YC:GR sites genome-wide in cancer mutagenesis. Thus, for SBSs at both CG:CG and YC:GR sites, we suggest a prominent role for oxidative damage by ROS and other electron-abstracting species.

One severe limitation in elucidating mechanisms of SBS is obviously the lack of information on the steady-state levels of modified bases and mismatches in cancer cells. Current efforts to address this critical issue [211,212,213,214,215] should at least temper these limitations, not only for the limited number of base modifications discussed here, but also for the much larger repertoire that may be formed by both endogenous and environmental agents, and which have not been addressed herein.
